# Offline and simulated-online fNIRS decoding of ankle movements for prospective exo-ankle control: a pilot study in healthy adults and ankle-impaired patients

**DOI:** 10.3389/fnrgo.2026.1726286

**Published:** 2026-10-16

**Authors:** Naqash Ahmad, Hammad Nazeer, Noman Naseer, Mirza Obaid Baig, Sadia Shakil, Kai-yu Tong

**Affiliations:** 1 Department of Mechatronics and Biomedical Engineering, Air University, Islamabad, Pakistan; 2 Faculty of Rehabilitation & Allied Health Sciences, Riphah International University, Islamabad, Pakistan; 3 Department of Biomedical Engineering, The Chinese University of Hong Kong, Hong Kong, Hong Kong SAR, China

**Keywords:** BCI, exo-ankle, fNIRS, gait rehabilitation, simulated online

## Abstract

This study developed and evaluated a non-invasive brain–computer interface (BCI) framework that decodes functional near-infrared spectroscopy (fNIRS) signals from the motor cortex to generate offline and simulated-online exo-ankle control commands, as a proof-of-concept step toward future prospective exo-ankle control. fNIRS data were recorded from 20 healthy participants and seven patients with ankle motor impairment using a PsychoPy-based paradigm of rest, dorsiflexion, and plantar flexion. Following the literature, after preprocessing, an evaluation of different combinations of six statistical features and four classifiers, namely, linear discriminant analysis (LDA), quadratic discriminant analysis (QDA), support vector machine (SVM), and k-nearest neighbor (kNN) was conducted. Under leave-one-subject-out (LOSO) cross-validation across 20 healthy participants, SVM achieved the mean offline classification accuracy of 72.50% ± 13.8% followed by kNN (65.83% ± 13.5%), LDA (63.75% ± 16.1%), and QDA (60.42% ± 17.7%). A Friedman test indicated a significant overall difference among classifiers (*p* = 0.0311). Simulated-online implementation used SVM with a sliding window approach with step sizes of 0.5, 1, and 1.5 s under participant-specific leave-one-trial-out validation. The results showed a highest mean accuracy of 62.2% ± 14.1% at a 1.5-s step size. The patient group showed a lower mean accuracy of 57.23% ± 5% using SVM under leave-one-trial-out cross-validation. Together, these offline and simulated-online results establish proof-of-concept feasibility for fNIRS-based exo-ankle control, providing a foundation for future prospective rehabilitation applications.

## 1 Introduction

The daily activities of humans are affected by so many factors like accidents, natural disasters, or health issues such as strokes or chronic illnesses. According to the study (Murray, 2022), approximately one in four adults are affected by severe musculoskeletal disorders globally, which often impact mobility. For the solution of these problems, exoskeletons are introduced. An exoskeleton is an assistive device that is worn externally to enhance the movements of the user. Additionally, they can be utilized to reduce the risk of injuries for those engaged in demanding tasks (Gutierrez et al., 2024). The ankle joint plays an important role in completing daily living activities, including support, mobility, thrusting forward, and absorbing shocks. Its main role is in weight bearing and facilitating mobility (Qiu et al., 2023). A study conducted in 2020 found that approximately 20% of adults in Australia had ankle-related problems (Al Mahrouqi et al., 2020). In the United States, approximately 25,000 people seek assistance every day for issues related to their ankles (Zhao Dubuc et al., 2022). Standing and walking difficulties are among the main effects of aging, trauma, accidents, and injuries. For example, more than 63% of stroke survivors have motor and cognitive deficits ranging from half-mild to severe (Crichton et al., 2016). While between 30% and 36% cannot walk without the assistance of a device (Kelly-Hayes et al., 2003). Due to this loss of independence, people face difficulty in participating in social and community activities. This further creates mental health issues (Winstein et al., 2016). Common motor deficits such as paralysis can be present in people with brain injuries, resulting in compensatory motions and asymmetric gait (Roelker et al., 2019). Exoskeletons can be helpful in this regard, to assist and provide therapy to overcome these problems. Initially, the passive exoskeletons were introduced. They use mechanical springs or joints to support and enhance the movements without external power. As technology progressed, active exoskeletons were developed. They use motors or batteries to assist or augment movement actively (Poliero et al., 2022). Over the years, ankle exoskeletons have progressed with technological advancements, such as the development of the ankle foot orthosis (AFO) (Shorter et al., 2011) which was then controlled by electromyography (EMG) using linear envelope techniques (Koller et al., 2015). A study conducted in 2018 examined the alterations in surface EMG data when walking with and without assistance from a myoelectric ankle foot orthosis (ankle exoskeleton). This research investigated factors, including module size and module timing. Investigating the dynamics of surface EMG data in relation to ankle exoskeletons has been made possible through the analysis of patterns related to ankle exoskeletons (Jacobs et al., 2018). Like ankle exoskeletons, EMG signals are also used for hand gesture classification as well (Ahmad et al., 2024).

Alongside EMG and electroencephalography (EEG), fNIRS is emerging as a promising new modality in BCI technology (Benerradi et al., 2023). fNIRS measures variations in the amounts of oxygenated hemoglobin (HbO) and deoxygenated hemoglobin (HbR) to learn more about brain activity (Kinder et al., 2022). Its primary benefits include mobility, safety, low noise, and ease of use. Since it is an optical imaging modality, its results are less vulnerable to electrical noise than those of EEG (Quaresima and Ferrari, 2019). The use of fNIRS as a neuroimaging tool has proven valuable in studies focused on motor control. While upper limb motor control has been extensively studied, the exploration of lower-limb motor control has been relatively limited mainly because lower-limb movements are inherently complex (Khan et al., 2021). However, recent studies have shown that fNIRS can be valuable in improving the understanding of lower-limb motor functions (Ahmad et al., 2025). A study combined fNIRS technology with a proportional derivative computed torque controller (PD-CTC) that includes gravity compensation. The goal is to reduce position errors during walking, demonstrating the potential of fNIRS for controlling hip and knee movements (Khan et al., 2018). The use of fNIRS systems allows us to classify the motor tasks and mental workload levels (Midha et al., 2021). Another study determined that there is a significant difference in HbO concentrations in the prefrontal cortex (PFC) and supplementary motor area (SMA) for chronic ankle patient group and healthy group (Luo et al., 2024). Another study utilized fNIRS signals to classify motion for a limb exoskeleton. The researchers employed the z-score method (Nazeer et al., 2020) to identify the channels out of a total of 12 channels (Nazeer and Naseer, 2022). A study was conducted using infrared spectroscopy to examine the activation of the cortex when individuals allocate executive resources during a motor task assisted by a robot (Le et al., 2022). An fNIRS-based cognitive motor action decoding framework is introduced (Akila et al., 2024) using fused wavelet, Hilbert, symlet, and Hjorth features with ICA preprocessing and LGBM. The classification achieved 98% accuracy for mental drawing and 97% for spatial navigation tasks. Recent studies also introduce some novel optimization approaches to enhance accuracy. A study (Akila et al., 2025) combines ETCKF preprocessing, temporal channel reconfiguration multi-graph CNN classification, and YDSEOA optimization and achieved higher HbO/HbR classification.

Despite significant advancements in exoskeleton technology and neuroimaging for motor control, there remains a gap in effective control methods for lower-limb applications, particularly for ankle movement. Previous studies have shown promising results using fNIRS and other neuroimaging tools for motor control; however, few have focused on optimizing control strategies specifically for gait phases, such as dorsiflexion and plantar flexion. This study uses classified data to generate prospective control commands for the exo-ankle, which are evaluated through offline classification and a simulated-online replay analysis of the system. A simulated-online analysis of the system is presented. Previous studies lacked patient involvement; this limitation is addressed in the present study by including patients with ankle motor impairment in the experimental evaluation. The research adheres to the BCI steps of data acquisition, preprocessing, feature extraction, classification, and implementation.

## 2 Materials and methods

A typical BCI system, shown in Figure 1, consists of five stages, which start with data acquisition, then preprocessing, feature extraction, classification, and finally, application of control commands generated.

**Figure 1:**
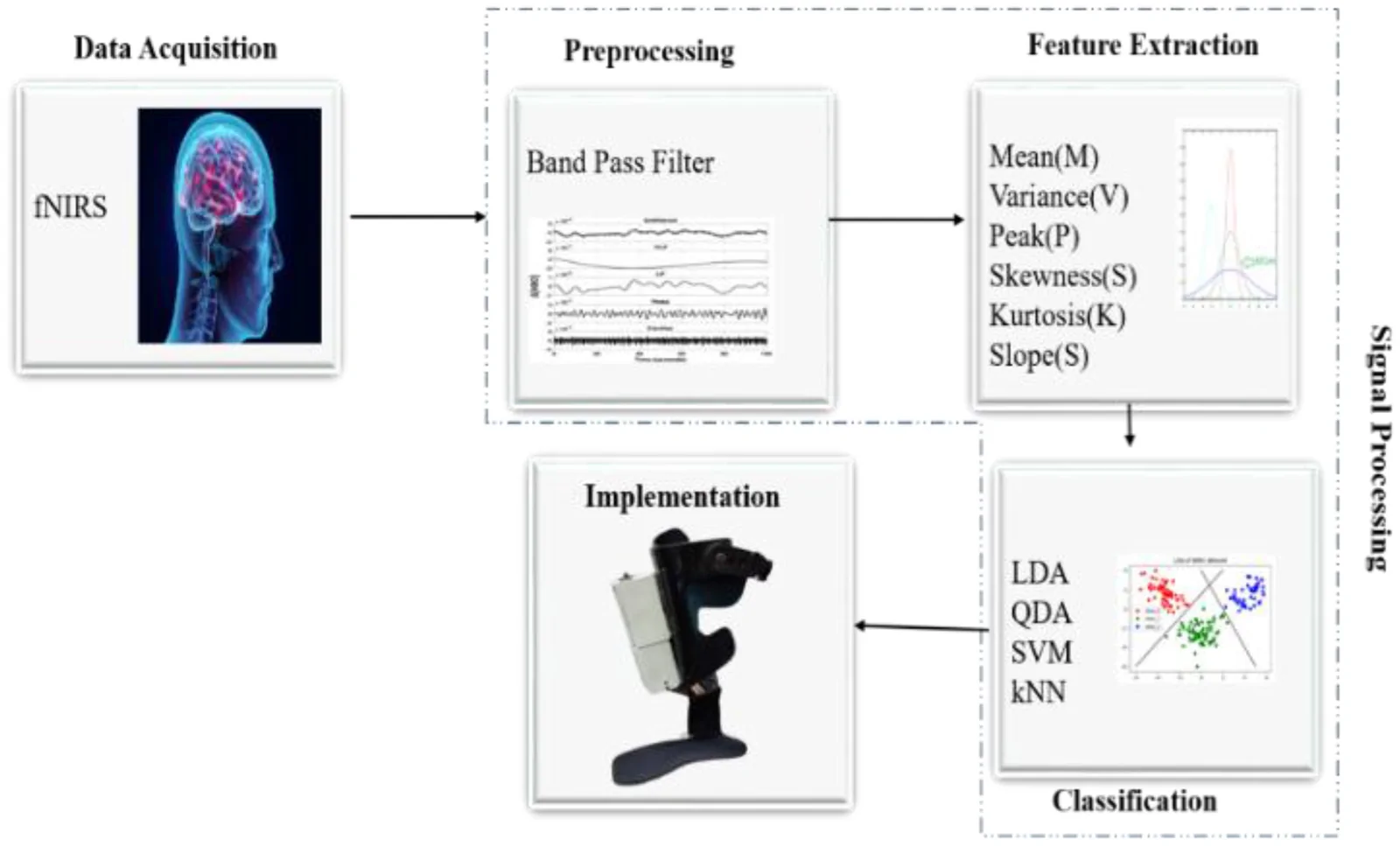
Steps of a BCI system showcasing the arrangement of steps from data acquisition to application.

### 2.1 Data Acquisition and ethics approval

Data were acquired from 27 participants with two groups, namely, a healthy and patient group. The healthy control group (*n* = 20) consisted of individuals including 10 male and 10 female individuals. Healthy participants were having no history of neurological disease. The average age of the participants was 23 ± 3 years. The patient group (*n* = 7) was comprised of individuals facing ankle mobility impairments due to various etiological factors.

Written informed consent was obtained from all participants prior to enrolment. Healthy participants were having no history of neurological disease, all of them were older than 18 years. While in the patient group, the subjects were clinically confirmed ankle mobility impaired with Berg Balance Score ≥ 40 for patient safety. This group included four male and three female subjects with an average age of 61 ± 12 years (see Table 1 for detailed demographics). For patients with hemiplegia, the affected side and therefore the contralateral hemisphere were determined clinically, based on the patient's documented diagnosis rather than by functional or imaging-based localization. Subjects with nicotine intake or those using medications that affect hemodynamics were excluded from the study. Participants involved in other studies or skin conditions at optode placement sites or inability to follow the experimental paradigm were also excluded.

**Table 1:** Demographic detail of patient group.

Participants	Gender	Age	Disease	BBS score
SUB 01	M	76	Diabetic neuropathy	44
SUB 02	M	46	Left hemiplegia	40
SUB 03	M	72	Ankle instability	40
SUB 04	M	60	Left hemiplegia	50
SUB 05	F	74	Left hemiplegia	42
SUB 06	F	40	Left hemiplegia	40
SUB 07	F	60	Left hemiplegia	48

fNIRS signals were recorded from the motor cortex (M1) in the contralateral hemisphere, while all participants engaged in a PsychoPy^®^-designed paradigm consisting of three ankle states: rest, dorsiflexion, and plantar flexion. Figure 2 shows the data acquisition montage for motor cortex. For data acquisition purposes, the NIRSport2 system (Gruszecka et al., 2020) was used. This system has a sampling rate of 10.1725 Hz and employs two discrete wavelengths of near-infrared light 760 nm and 850 nm. These wavelengths correspond to the absorption spectra of HbR and HbO, respectively. The source–detector separation was 3 cm, standard for NIRSport2 adult probe configurations. Optode placement followed the international 10–20 system, with the 20-channel montage covering the primary motor cortex. Prior to each recording, channel signal quality was verified using the Aurora acquisition software's signal-quality indicator, and data acquisition began only once all channels displayed acceptable coupling. As all retained channels had already passed this pre-recording quality check, no channels or participants were excluded *post-hoc* during analysis.

**Figure 2:**
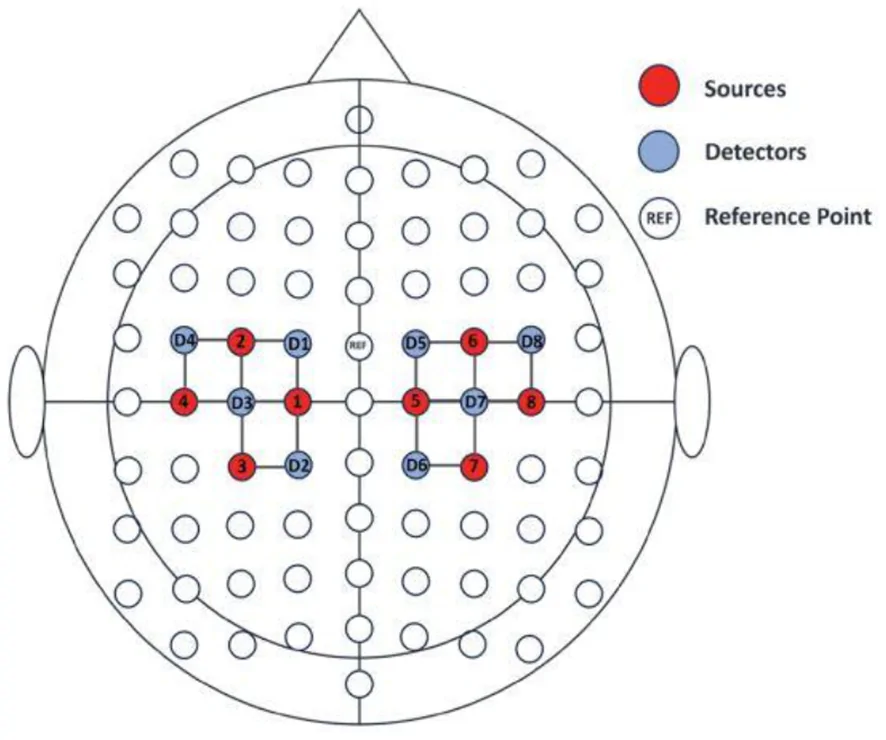
Data acquisition using NIRSport2, 20 channel data acquisition with 8 × 8 montage for motor cortex taken from AURORA software.

This study used an 8 × 8 emitter–detector montage covering the primary motor cortex (AlQahtani et al., 2024). This montage covers the major M1 area but does not include the medial Cz and FCz regions, which correspond to the lower-limb representation in the motor homunculus. As a result, the classified signal cannot be confirmed to originate specifically from lower-limb M1 activity; it may also reflect broader cortical, systemic, or movement-related contributions. The research strictly followed the guidelines stated.

### 2.2 Experimental paradigm

The experimental paradigm was designed to record fNIRS signals during alternating periods of ankle movement and rest. For healthy participants, the protocol lasted 180 s, beginning and concluding with a 45-s rest period; the intermediate block consisted of three cycles of a 5-s movement task (dorsiflexion/plantar flexion) followed by a 10-s rest, as illustrated in Figure 3. The healthy subject's data were acquired without wearing or using exo-ankle. For patients, a modified paradigm, shown in Figure 4, was employed to accommodate their specific needs (Caliandro et al., 2020). This starts with 45-s initial rest period. The activity block was similarly adjusted, with movement tasks extended to 15 s, each followed by a 10-s rest period and ends with 45-s final rest. This concluding rest period resembles the initial resting time to be strategically incorporated to provide participants with enough time to recover (Quaresima and Ferrari, 2019). During each activity block, the exo-ankle mechanically assisted the patient in performing the dorsiflexion and plantar-flexion movements, rather than the patient generating the movement unassisted.

**Figure 3:**
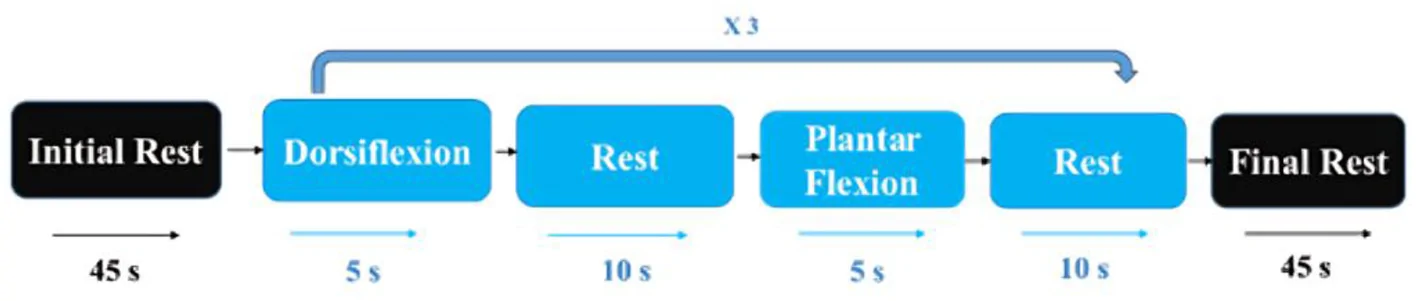
Experimental paradigm of 180 s for healthy group having consecutive activity and rest.

**Figure 4:**
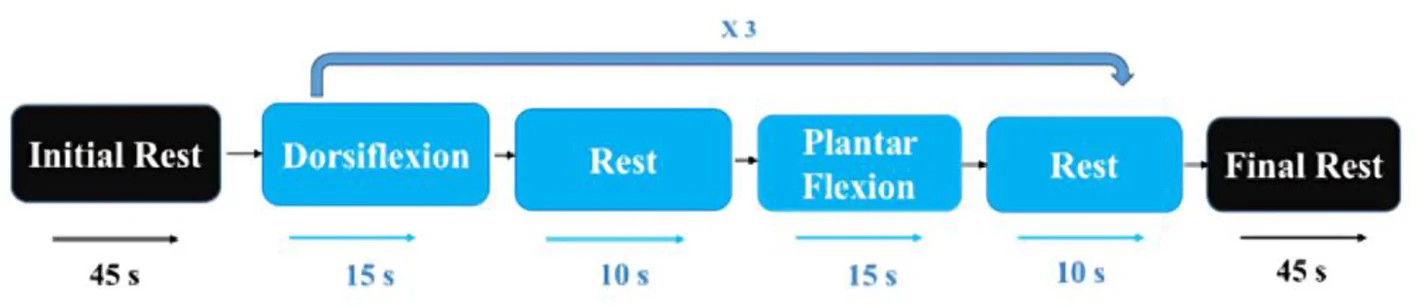
Experimental paradigm for patient group of 240 s having consecutive activity and rest.

To design this experimental paradigm, the software PsychoPy^®^ was used. PsychoPy^®^ is a flexible software package whose source has been used in various studies focusing on time-based event strategies, particularly in tasks related to replicating specific movements. Researchers have made use of the capabilities of PsychoPy^®^ to create experiments where participants are shown stimuli at predetermined time intervals.

For the patient cohort, the longer activities were implemented to provide a sustained neural stimulus, thereby amplifying the haemodynamic signal to better overcome a potentially attenuated or delayed response.

### 2.3 Signal acquisition and preprocessing

To acquire the meaningful signal from raw fNIRS data, the modified Beer–Lambert law (MBLL) (Chen et al., 2020) is used. This method transformed the raw optical density signals into changes in the concentrations of HbO and HbR. These changes are denoted as ΔC_HbO_ (t) and ΔC_HbR_ (t). The transformation was done using an expression:


$$\begin{array}{c} \left[\begin{array}{c} \Delta C_{HbO}\left(t\right)\\ \Delta C_{HbR}\left(t\right) \end{array}\right]=\;\frac{{\left[\begin{array}{cc} \alpha_{HbO}\left(\lambda_{1}\right) & \alpha_{HbR}\left(\lambda_{1}\right)\\ \alpha_{HbO}\left(\lambda_{2}\right) & \alpha_{HbR}\left(\lambda_{2}\right) \end{array}\right]}^{-1}\left[\begin{array}{c} \Delta A\left(t,\lambda_{1}\right)\\ \Delta A\left(t,\lambda_{2}\right) \end{array}\right]\;}{d^{*}l} \end{array}$$ (1)

where *l* is used to represent the distance between the source and detector, and *d* represents the factor that accounts for the curved path length. The terms ΔA(t,λ_1_) and ΔA(t,λ_2_) stand for the absorption at two moments, while α_HbR_ (λ_1_) and α_HbO_ (λ_2_) represent the extinction coefficients HbR and HbO measured in [μM^−1^cm^−1^].

Twenty channels of HbO were analyzed. Signals were passed through a band-pass filtered between 0.01 and 0.30 Hz using a fourth-order Butterworth filter applied in both forward and reverse directions (zero-phase), attenuating instrument drift below the passband and cardiac and respiratory components above it while preserving the task-evoked haemodynamic response. No additional motion-artifact detection or correction procedure was applied.

To account for the haemodynamic response delay, the event labels were shifted forward by 3 s relative to the recorded signals before segmentation. The initial 45-s rest period and the additional 3-s buffer (approximately 491 samples at 10.1725 Hz) were excluded from classification, and the shifted labels were used to assign the remaining data to dorsiflexion, plantar-flexion, and rest blocks. For each channel, the mean and standard deviation of this base line interval were computed, and the full recording was expressed as z-scores relative to that baseline. The baseline interval was used solely for normalization and was excluded from classification, so no task-related data and no data from any other participant contributed to the normalization of a given recording.

Before evaluating classification performance, we verified that the recorded fNIRS signal shows a task-locked haemodynamic response consistent with movement-evoked cortical activity. Figure 5 shows the grand-average HbO and HbR time courses across the full experimental paradigm for the healthy cohort, using the 3-s delay. These traces are shown prior to the band pass filtering applied for feature extraction, so they represent the raw, unfiltered HbO/HbR signal. HbO and HbR show a largely anti-correlated, task-locked modulation, with HbO rising and HbR falling during several of the dorsiflexion and plantarflexion blocks.

**Figure 5:**
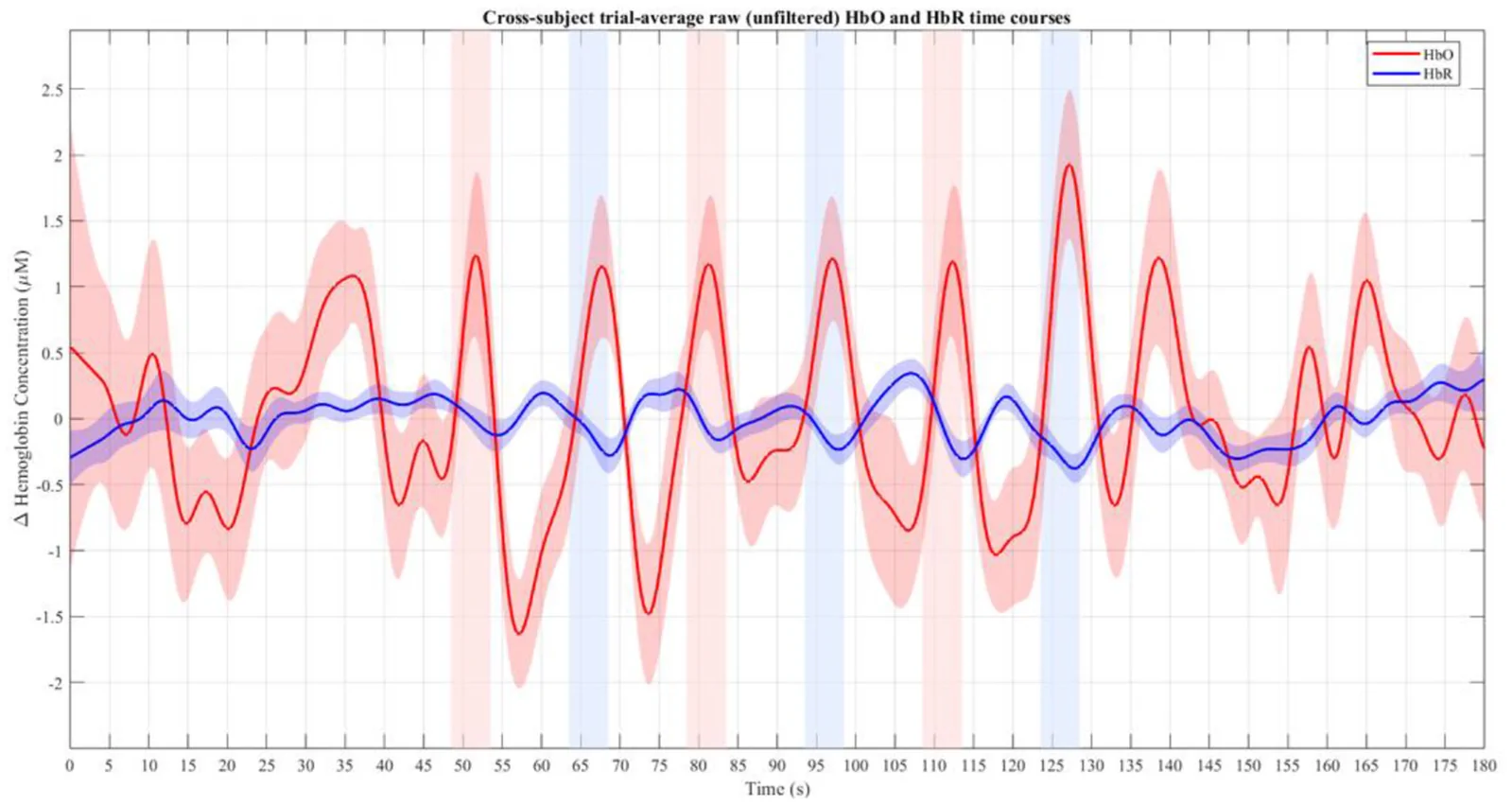
Cross-subject trial-average raw (unfiltered) HbO (red) and HbR (blue) time courses across the full experimental paradigm for the healthy cohort (mean ± SEM shading).

### 2.4 Feature extraction

A feature is an individual measurable property or characteristic used to describe data. Features are extracted from filtered data to simplify and enhance the learning process by providing relevant information for models to identify patterns and make predictions more accurately. Following the literature (Shabbir Minhas et al., 2024), six statistical features were selected, including mean, variance, skewness, kurtosis, peak, and slope.

HbO signals were processed on a channel-by-channel basis rather than averaged across the montage: for each block of activity or rest, the selected statistical features were computed separately for each of the 20 HbO channels. Accordingly, an “N-feature combination” (e.g., the six-feature combination) refers to the number of statistical measures included, not to the dimensionality of the final feature vector.

#### 2.4.1 Mean

The mean, denoted as *M*, represents the central tendency of the signals. It provides valuable information about the baseline or typical levels of activation.


$$\begin{array}{c} M=\frac{1}{N}\overset{N}{\underset{i=1}{\sum }}Z_{i} \end{array}$$ (2)

In this case, *N* refers to the count of observations, while Z_i_ represents the change for each observation.

#### 2.4.2 Variance

Variance denoted by *V* represents the extent to which data are spread out or dispersed. It helps us understand how much the signal values differ from the average, giving insights into the data's characteristics and patterns.

The level of brain activation shows a range of variability. When there is variance, it means that there is a range of signal intensities, which indicates increased variability in the way the brains respond.


$$\begin{array}{c} V=\frac{\sum_{i=1}^{n}{\left(X_{i}-\bar{X}\right)}^{2}}{n-1} \end{array}$$ (3)

where *n* represents the number of data points, *Xi* denotes each value in the data set, and *X* represents the value.

#### 2.4.3 Skewness

Skewness, represented by S, measures the degree of unequal signal distribution. Positive skewness denotes the presence of a tail in the distribution, indicating the presence of stronger signals. On the other hand, if skewness is negative, it indicates a tail and on the left side indicates weaker signals. Skewness helps us understand the form and balance of the distribution.


$$\begin{array}{c} S=E\left[{\left(\frac{Z-u}{\sigma }\right)}^{3}\right] \end{array}$$ (4)

The standard deviation of variable *Z* is denoted as σ, and the expected value of *Z* is denoted as *E*, where *u* is the mean value in this context.

#### 2.4.4 Kurtosis

Kurtosis is indeed a measure of the “tailedness” of a distribution, meaning it describes the presence and extremity of outliers. A high kurtosis indicates that the distribution has more pronounced tails, suggesting more extreme outliers or signal intensities far from the mean. A low kurtosis implies that the distribution has lighter tails, with fewer extreme outliers, and the data points are generally more concentrated around the mean.


$$\begin{array}{c} K=E\left[{\left(\frac{Z-u}{\sigma }\right)}^{4}\right] \end{array}$$ (5)

The kurtosis of variable *Z* is denoted as σ, and the expected value of *Z* is denoted as *E*, where *u* is the mean value in this context.

#### 2.4.5 Slope

In the context of time series data analysis, the slope represented by S denotes the rate of change of a variable concerning time. It is calculated as the ratio of the change in the variable's value to the change in time. The equation of a line having slope m can be defined as


$$\begin{array}{c} y=mx+b \end{array}$$ (6)

where *m* represents the slope, and in the case of fNIRS, the slope provides insight into the temporal dynamics of haemodynamic responses, indicating whether HbO levels are increasing or decreasing and at what rate.

#### 2.4.6 Peak

A peak represented by P refers to the point or the maximum value in a distribution, signal, or dataset. As a feature, it captures extreme values, helping identify critical patterns in areas such as time series analysis, signal processing, or anomaly detection. Peaks are often used to highlight significant events or outliers in the data.

## 3 Classification

Each healthy participant's 180-s recording yields approximately 1,800–1,850 raw time points at the system's 10.1725 Hz sampling rate, but one classifier sample does not correspond to a single time point. One classifier sample is one block either movement or rest. Each healthy participant contributed 12 blocks including, three dorsiflexion, three plantar flexion, and six rest, giving 240 samples across the 20 participants (120 rest, 60 dorsiflexion, and 60 plantar flexion). Only HbO channels were utilized in this study. This decision was based on the consistently higher SNR of HbO relative to HbR in motor task-based fNIRS paradigms (Huang et al., 2026). After removing the initial and final rests, the data are subjected to feature extraction.

Model performance was estimated using LOSO cross-validation across the 20 participants. In each of the 20-fold, the model was trained on 19 participants and evaluated on the entirely held-out participant, so that no sample from a test participant contributed to model fitting at any stage. Feature parameters were selected on the training participants only and applied unchanged to the held-out participant. All reported metrics derive exclusively from held-out predictions.

LOSO was adopted in preference to random k-fold partitioning of individual time points. Because consecutive time points' data within a movement block are strongly auto-correlated, random assignment to folds places highly correlated observations from the same block in both training and test sets, which inflates apparent performance. LOSO removes this possibility by construction and additionally quantifies the more demanding and clinically more relevant case of generalization to an unseen individual.

Four classifiers were compared: LDA, QDA, SVM, and kNN.

Within each LOSO fold, the number of neighbors k for kNN was selected by grid search using 5-fold cross-validation applied to the training data of that fold only. The held-out participant contributed to neither hyperparameter selection nor model fitting. Selected values varied across folds, with a mean of 3.8. The remaining classifiers used fixed settings applied identically across all folds: LDA used a linear discriminant, and QDA used a diagonal quadratic discriminant. For the SVM classifier, a linear kernel was used, and the box-constraint parameter (C) was selected using grid search within the training data of each cross-validation fold. The mean selected value of (C) across the folds was 0.7. All analyses were performed in MATLAB R2020a (The MathWorks, Natick, MA, USA). A fixed random seed [rng(42)] was set prior to all cross-validation and bootstrap procedures to ensure reproducibility of the reported results.

### 3.1 LDA

LDA is commonly used for dimensionality reduction and classification. Its main goal is to maximize the between class variance while minimizing the variation within each class.

The core principle behind LDA is to find out a transformation of the input features that optimizes class separation (Suesse et al., 2023). To start with, LDA calculates scatter matrices within classes (Equation 7) and between classes (Equation 8). These represent the variation within each class and measuring the separation between class means.


$$\begin{array}{c} S_{\omega }=\overset{C}{\underset{i=1}{\sum }}\overset{n_{i}}{\underset{j=1}{\sum }}{\left(x_{ij}-u_{i}\right)\left(x_{ij}-u_{i}\right)}^{T} \end{array}$$ (7)

$$\begin{array}{c} S_{b}=\overset{C}{\underset{i=1}{\sum }}n_{i}\left(u_{i}-u\right){\left(u_{i}-u\right)}^{T} \end{array}$$ (8)

where C is the number of classes in the data, n_i_ is the at a point in the i-th class, x_ij_ is the j-th data point in the i-th class, and μ_i_ is the average of i-th class.

### 3.2 QDA

QDA uses quadratic function to draw boundaries between classes. It allows each class to have its covariance matrix. It is more flexible than LDA for handling data with varying class distributions. QDA is particularly useful when class variances differ significantly.

In QDA, each class is modeled using a multivariate normal distribution. Unlike LDA, every class has its own mean vector (μ) and covariance matrix (Σ), resulting in quadratic decision boundaries.


$$\hat{k}=(\arg\left(-\frac{1}{2}\ln\left|\Sigma_{k}\right|-\frac{1}{2}{\left(x-\mu_{k}\right)}^{\text{T}}\Sigma_{k}^{-1}\left(x-\mu_{k}\right)\right)+\ln\pi_{k})$$ (9)

where x is the feature vector to classify, μ_*k*_ is the mean vector of class k, Σ_*k*_ is the covariance matrix of class k, |Σ_*k*_| is the determinant of the covariance matrix Σ_*k*_, Σ_*k*_^1^ is the inverse of the covariance matrix Σ_*k*_, and π_*k*_ is the prior probability of class k. The decision boundaries created by QDA are more flexible than those of LDA, allowing for the capture of relationships in the data. This flexibility comes at the cost of increased model complexity and potential sensitivity (Wu and Hao, 2022).

### 3.3 SVM

SVM is used for both classification and regression tasks. Its primary goal is to identify a hyperplane that effectively separates different classes. It tries to maximize the distance between these classes (Rakhmetulayeva et al., 2018). This distance is known as the margin and is defined by specific data points from each class. These data points are referred as support vectors. SVM can employ kernel functions to transform the input data into a higher-dimensional space when class boundaries are not linearly separable. In this study, a linear kernel was used.


$$\begin{array}{c} f\left(x\right)=sign\;\left(\overset{n}{\underset{i=1}{\sum }}a_{i}y_{i}x_{i}^{T}x+b\right) \end{array}$$ (10)

where xi are the support vector (training data points closest to the hyper plane), ai are the Lagrange multiplier obtained from the optimization process, yi is the label of the support vector (y_(i )ε {−1,1}), x is the input feature vector to be classified, and b is the bias term (determined during the optimization).

### 3.4 kNN

kNN is a simple, non-parametric classification algorithm that assigns a data point to the most common class among its neighbors. It works based on distance metrics, such as Euclidean distance. It is effective for tasks where similar instances have similar labels. kNN is easy to implement but can be computationally expensive with large datasets.

Feature space can be represented as follows:


$$\begin{array}{c} d\left(X_{i},X_{j}\right)=\sqrt{\overset{n}{\underset{P=1}{\sum }}{\left(X_{i,P}-X_{j,p}\right)}^{2}} \end{array}$$ (11)

where X_i, p_ denotes the *p*-th feature (or coordinate) of point X_*i*_, and X_j, p_ denotes the *p*-th feature (or coordinate) of point X_*j*_. Another advantage is that kNN is considered a non-parametric algorithm, which means it does not rely on specific assumptions about the data distribution. This makes it quite flexible and adaptable to scenarios. The selection of the number of neighbors commonly represented as “k” in the kNN algorithm significantly affects the performance of the model. To find the value for the “*k*” parameter in the kNN algorithm, grid search method is used. It works by graphing the model's performance, such as accuracy or error rate, against different “*k”* values. This method allows us to strike a balance between capturing patterns in the data and avoiding overfitting.

## 4 Offline application and simulated-online analysis

Simulated-online replay involves using pre-recorded fNIRS data from the subject to generate prospective control commands within a controlled, offline setting. In this context, “simulated online” refers to the offline connection between pre-recorded fNIRS data and the exo-ankle device's command interface, resulting in a virtual real-time replay rather than prospective, hardware-actuated control.

A 25-point (≈2.5 s) window was used with SVM for this offline classification, with a 5-point (≈0.5 s) step between consecutive windows. This overlapping technique ensures smooth transitions between windows, allowing continuous data evaluation. The step size was subsequently changed to 10 data points (≈1 s) and 15 data points (≈1.5 s) to identify the highest performing step size for this proof-of-concept decoding approach. To avoid windows containing a mix of activity and rest labels and to avoid the data leakage, any window spanning the boundary between two experimental phases was excluded from analysis. This captures the prevailing trend or dominant behavior in the decoded output within that time frame, illustrating how such decisions could inform prospective exo-ankle control, without evaluating or measuring any resulting device or joint movement.

A window size of 25 data points (≈2.5 s at the system's 10.1725 Hz sampling rate) was selected based on prior fNIRS-BCI work using similar smaller window durations for real-time command generation (Khan et al., 2018) consistent with the broader 2- to 7-s range reported as optimal for fNIRS classification (Hong et al., 2015) and short enough to fit within the 5-s activity blocks of the experimental paradigm. As the present study was evaluated offline without physical exo-ankle hardware, the additional latency from command generation and mechanical actuation could not be measured.

## 5 Results for healthy group

The study explores the best among the tested set feature combination and classifier for the ankle rehabilitation BCI system. The discussion is based on different evaluation metrics such as accuracy, F1 score, precision, and recall.

### 5.1 Feature combination comparison

Six statistical measures are used as features, such as peak, mean, variance, skewness, kurtosis, and slope. Additionally, two-, three-, four-, five-, and six-feature combinations were utilized to estimate classification accuracy and other performance measures to identify the best among the tested set feature combination. For this purpose, SVM is used as the primary classifier.

Initially, the feature combinations were tried on healthy participants, and then the highest-performing combination was used to get the results for patients' group. The combination of six features (peak, mean, variance, skewness, kurtosis, and slope) demonstrates the highest accuracy of 72.5%. Following closely, the five-feature combination (mean, peak, skewness, kurtosis, and slope) and four-feature combination (mean, variance, peak, and slope) yielded classification accuracy of 71.6% and 70.1%, respectively.

The extensive examination carried out on combinations of features uncovers a pattern; combinations that include the “skewness” feature show relatively lower accuracy, while those that involve the “slope” attribute demonstrate higher accuracy when classifying tasks using fNIRS signals. This observation leads to the conclusion that the “slope” as associated with higher accuracy in the evaluated combinations.

### 5.2 Classifier comparison

#### 5.2.1 Accuracy

Three classifiers were included to validate the observations made from SVM and to get the best classifier. The results support the observations for best among the tested set feature combination and give a complete picture of classification results.

The performance of LDA, QDA, kNN, and SVM under LOSO cross-validation is compared in Figure 6 and summarized in Table 2. SVM achieved the highest accuracy at 72.50%, followed by kNN (65.83%), LDA (63.75%), and QDA (60.42%). All four classifiers exceeded the 50.0% majority-class baseline, and balanced accuracies ranged from 55.00% to 68.06% against a chance level of 33.3% for three-class discrimination. Considerable variability was observed across participants reflecting the between-participant differences in cortical anatomy, optode coupling, and signal quality that a participant-independent model must accommodate.

**Figure 6:**
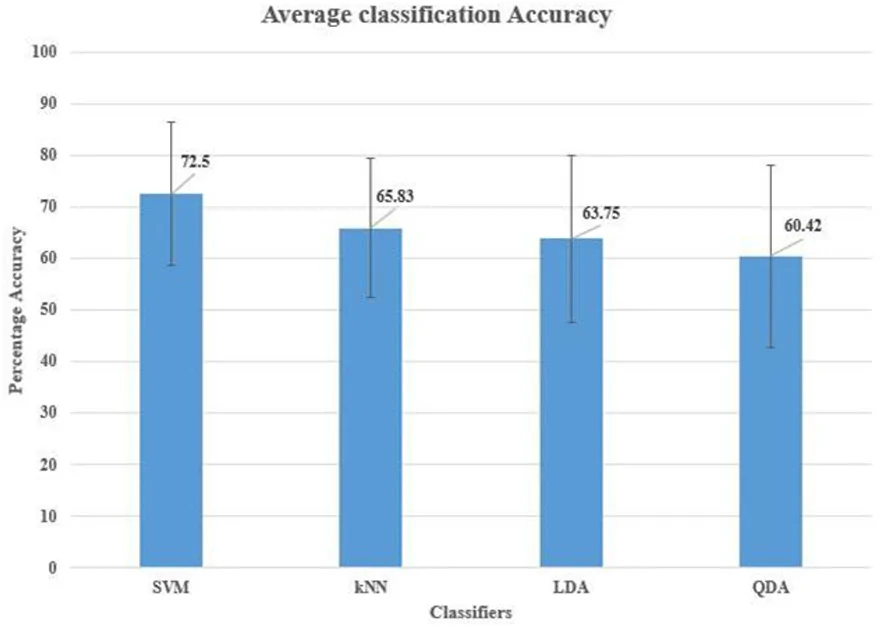
Classifier comparison between four machine learning classifiers with their standard deviation under LOSO.

**Table 2:** Outcome measures.

Classifier	Precision (%)	Recall (%)	F1 score (%)
LDA	64.66	63.75	64.14
SVM	72.15	72.50	72.28
KNN	62.24	65.83	62.20
QDA	64.49	60.42	62.00

To quantify the influence of the validation scheme, the same features and classifiers were also evaluated using the partitioning employed in an earlier version of this analysis, in which individual observations were assigned at random to 10 cross-validation folds. Under that scheme, apparent accuracy reached 93.13%. Because consecutive observations within a movement block are strongly auto-correlated, random assignment places highly correlated observations from the same block in both the training and test partitions, so the classifier is effectively evaluated on data closely resembling its training set.

Under LOSO validation, which excludes this possibility by construction, accuracy was 72.50%. The difference of 20.6 percentage points indicates the magnitude of inflation that observation-level partitioning can introduce in continuous fNIRS recordings. The LOSO figure is reported throughout as the valid estimate of generalization performance.

Classification performance was further evaluated at the subject level. The mean accuracy was 63.7% ± 16.1% for LDA, 72.5% ± 13.8% for SVM, 65.83% ± 13.5% for kNN, and 60.4% ± 17.7% for QDA. The corresponding t-based 95% confidence intervals were 56.23%−71.27% for LDA, 66.04%−78.96% for SVM, 59.5%−72.2% for kNN, and 52.13%−68.70% for QDA. These intervals quantify the uncertainty around the observed mean subject-level accuracies across participants.

Participant-level accuracies were compared using the Friedman test, with Kendall's W reported as the effect size. Pairwise *post-hoc* comparisons used the Wilcoxon signed-rank test with Bonferroni correction for the six pairwise comparisons (α = 0.0083). The significance threshold for the omnibus test was α = 0.05. The Friedman test was significant (*p* = 0.0311), but no pairwise comparison survived Bonferroni correction (all *p* > 0.0083; Table 3), including SVM vs. LDA (*p* = 0.0087), which falls just short of the corrected threshold.

**Table 3:** *Post-hoc* pairwise comparisons of participant-level classification accuracy using the Wilcoxon signed-rank test with Bonferroni correction.

Classifier pair	*p-value*
SVM and LDA	0.0087
SVM and QDA	0.0141
SVM and kNN	0.0288
kNN and QDA	0.4390
LDA and kNN	0.9714
LDA and QDA	0.5557

Pairwise *post-hoc* comparisons using the Wilcoxon signed-rank were used to test the significance as shown in Table 3.

Chance-level performance was additionally estimated using a permutation test, in which class labels were randomly shuffled within each participant and classification accuracy recomputed across 500 permutations. This gives a more accurate estimate of chance than the nominal 33.3% three-class baseline since it accounts for the unequal number of rest, dorsiflexion, and plantar flexion samples. Mean permutation-based chance accuracy was 34.9% for LDA, 37.0% for SVM, 45.5% for kNN, and 34.1% for QDA. All four classifiers exceeded their respective chance distributions (*p* < 0.002 in each case, the resolution limit of a 500-permutation test), confirming that observed accuracies of 63.75% for LDA, 72.50% for SVM, 65.83% for kNN, and 60.42% for QDA were statistically significant. These results indicate that the observed decoding performance was above the participant-wise permutation distributions.

#### 5.2.2 Confusion matrix

Figure 7 shows the confusion matrices obtained under LOSO cross-validation for all four classifiers, together with the corresponding per-class recall and precision. All four classifiers separated rest from movement more reliably than they separated the two movement directions from one another.

**Figure 7:**
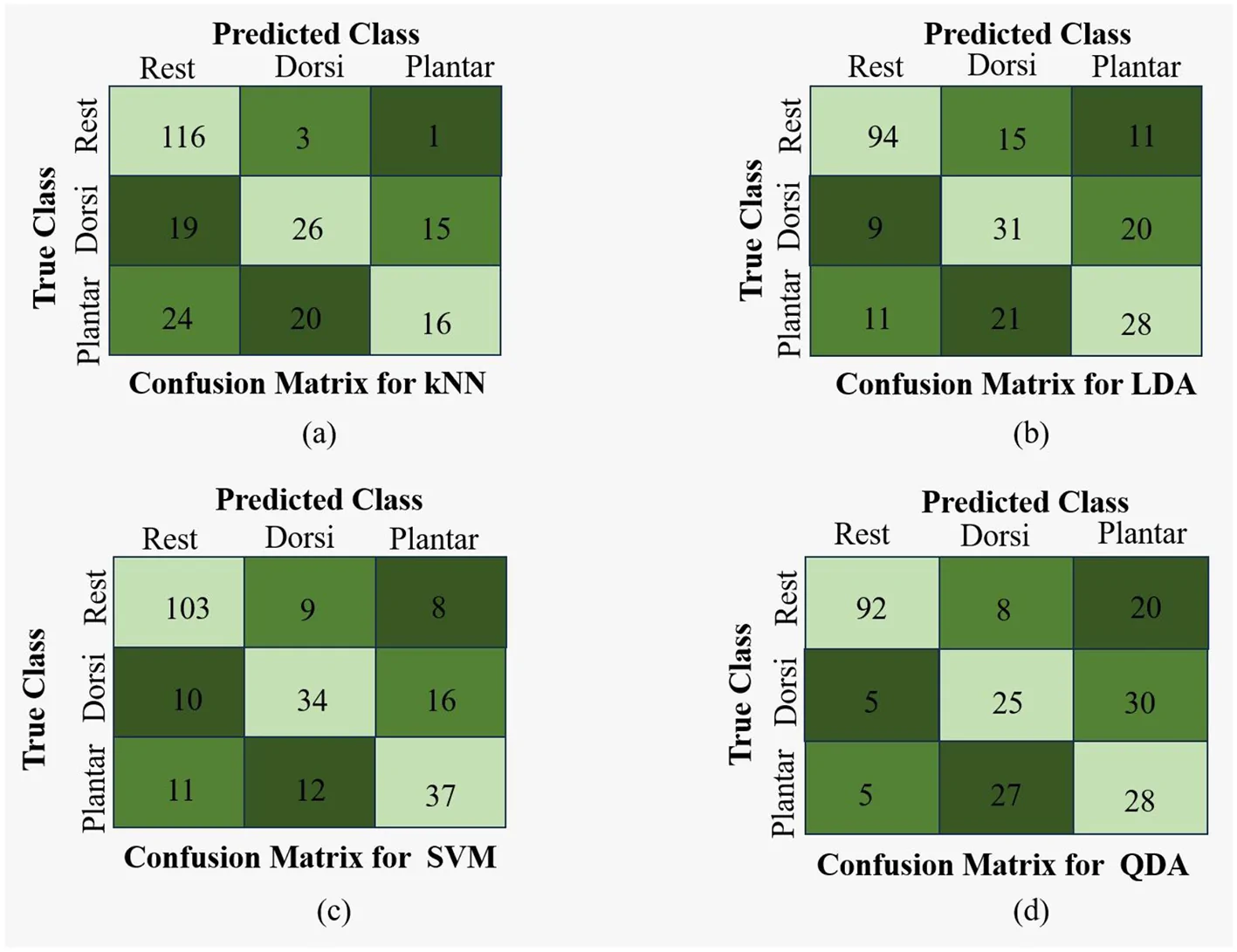
Confusion matrices for classifiers representing **(a)** kNN, **(b)** LDA, **(c)** SVM, and **(d)** QDA, respectively.

#### 5.2.3 Precision

Precision measures how well a model's positive predictions are actually correct. It is computed by dividing the number of true positive predictions by the total number of true positive and false positive predictions.


$$\begin{array}{c} Precision=\frac{True\;Positive}{\;True\;Positive+False\;Positive} \end{array}$$ (12)

Weighted precision was highest for SVM at 0.72, followed by LDA (0.65), QDA (0.64), and kNN (0.62). This indicates that SVM identified true positives while producing comparatively fewer false positives.

#### 5.2.4 Recall

Recall, referred to as sensitivity or true positive rate, is a parameter used in classification issues that assesses a model's capacity to accurately choose all relevant instances from the total number of real positive examples in the dataset. It is computed by dividing the number of true positive predictions by the total number of true positive and false negative predictions.


$$\begin{array}{c} Recall=\frac{True\;Positive}{True\;Positive+False\;Negative} \end{array}$$ (13)

Weighted recall, which equals overall accuracy in a single-label multiclass setting, was 0.73 for SVM, 0.65 for kNN, 0.63 for LDA, and 0.60 for QDA.

#### 5.2.5 F1 score

The F1 score aggregates recall and precision into a single number. It is the harmonic means of recall and precision, offering a ratio of the two measures. The following formula is used to determine the F1 score:


$$\begin{array}{c} F1\;Score=2\;x\;\frac{Recall\;X\;Precision\;}{Recall+Precision} \end{array}$$ (14)

The F1 score indicates better balance between precision and recall. Weighted F1 was 0.72 for SVM, 0.64 for LDA, 0.62 for kNN, and 0.62 for QDA. Macro F1, which weights the three classes equally regardless of their sample counts, was 0.68 for SVM, 0.59 for LDA, 0.54 for kNN, and 0.55 for QDA. Table 2 shows weighted values of accuracy precision and recall.

### 5.3 Simulated online

A prefabricated exo-ankle (Yeung et al., 2021) is used for practical implementation. Figure 8 shows a different view of exo-ankle used in this study. The device includes a stepper motor with a precision of 0.8 degrees per step, weighs 0.5 kg, and integrates a gear mechanism for torque transfer, a servo motor, and a force sensing resistor (FSR). Its main purpose is to assist with movement in individuals with impaired ankle function, acting as a power-assisted ankle robot (PAAR). Alternatively, it can passively support individuals with a dropped foot by locking and releasing the ankle joint to ensure foot clearance during the swinging phase. In this mode, it functions as a swing-controlled ankle robot (SCAR).

**Figure 8:**
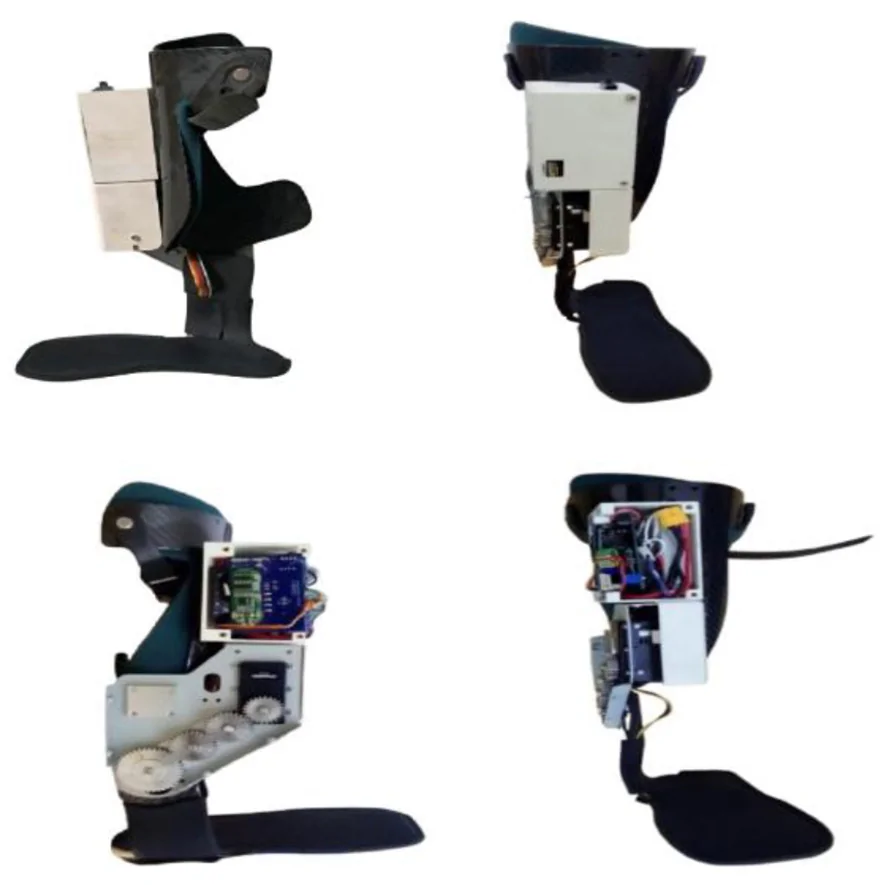
Different views of hardware of exo-ankle used in this study.

The simulated-online analysis used a sliding window of data points (2.5 s) advanced in steps of 5, 10, or 15 data points (0.5, 1.0, and 1.5 s, respectively). A 5-data point step leaves 20 data points shared between consecutive windows (80% overlap), a 10 data points step leaves 15 shared data points (60% overlap), and a 15-data point step leaves 10 shared data points (40% overlap). The step size determines the command update interval: the predicted ankle state is issued once per step, giving update intervals of 0.5, 1.0, and 1.5 s. The simulated-online analysis used leave-one-trial-out validation within each participant: for each of the three trials in turn, the SVM classifier was trained on the remaining two trials of that participant and tested on the held-out trial, with the reported accuracy averaged across the three rotations. The held-out trial contributed no data to the model evaluating it. This participant-specific scheme corresponds to a system calibrated on a short recording session from the individual user.

### 5.4 Results for patient group

Using the SVM algorithm as the classifier and a selected set of six features, the classification accuracies for individual patients are presented in Figure 9. The approach yielded an average accuracy of 57.23 ± 5 under leave-one-trial-out cross-validation. The healthy and patient cohorts differed systematically in age (23 ± 3 vs. 61 ± 12 years), movement-block duration (5 s vs. 15 s), diagnosis, and prior device exposure, and the patient group is small and diagnostically heterogeneous. Given these differences, the two cohorts are not methodologically comparable. Patient activities were assisted by the exo-ankle, while healthy subjects performed unassisted, so any accuracy gap cannot be attributed to a single cause.

**Figure 9:**
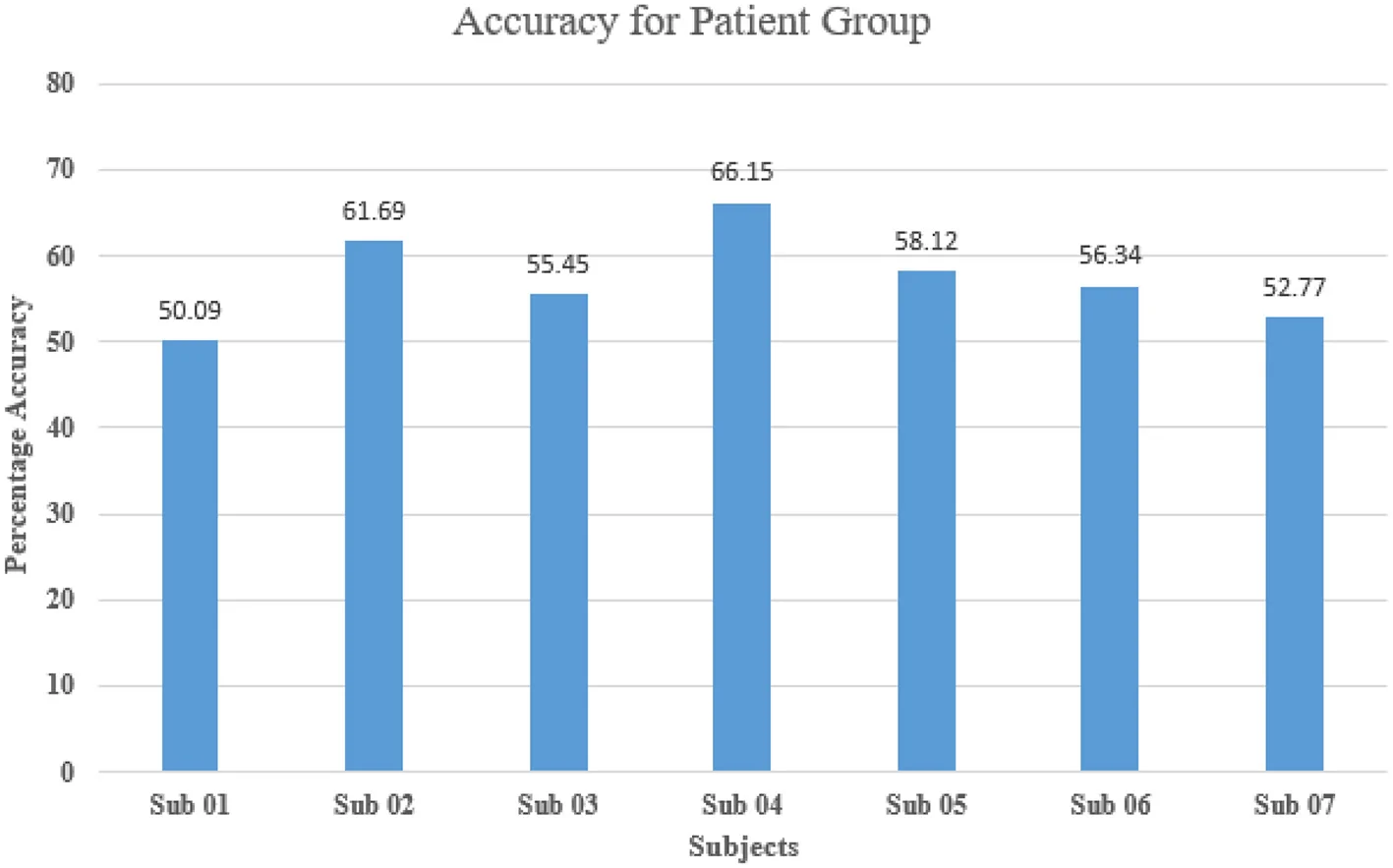
Classification accuracies of patient group using six feature combination and SVM under leave-one-trial-out cross-validation.

## 6 Discussion

This study presents an offline and simulated-online decoding approach toward prospective exo-ankle control using fNIRS signals. It identifies the highest-performing feature combination and machine learning algorithm. The system is tested using a simulated-online approach on different window sizes to obtain best among the tested set step size. The research follows the standard BCI methodology, encompassing data acquisition, preprocessing, feature extraction, classification, and implementation. The study specifically targets three gait cycle states: rest, dorsiflexion, and plantar flexion.

In previous studies, a comparison between different machine learning classifiers was presented as a study (Khan et al., 2018) comparing ML algorithms on fNIRS data for ankle motions and suggested that SVM results better than other ML classifiers with an average of 75% accuracy. Another study (Hasan et al., 2020) compared the ML classifiers for fNIRS data and reported random forest as the best among the tested set classifier with an accuracy of 92.5%. A study on frontal cortex haemodynamic responses during cognitive tasks using wavelet transforms (Abibullaev and An, 2012) finds out that the performance of a classifier is based on input data and their quality. A comparison between LDA and SVM was presented (Hu et al., 2012) where SVM emerges as performing better in compared classifier set for imagery rotation task (96.28%). In the past, highest performing feature combination using Fuzzy combination (Singh et al., 2018) was determined.

This study extensively compared four ML classifiers: LDA, QDA, SVM, and kNN. For classification purposes, best among the tested set feature combination was determined; the previous studies were restricted to only two-dimensional and three-dimensional feature combinations. The proposed methodology in our research explores four-, five-, and six-dimensional feature combinations. The study indicates that the combination of mean, variance, kurtosis, peak, skewness, and slope achieved the highest accuracy at 72.50% using SVM under LOSO cross-validation. This is lower than accuracies reported in several earlier fNIRS studies, which generally employed k-fold partitioning of individual observations rather than participant-independent validation. The present result should therefore not be read as poorer decoding but as a more conservative estimate obtained under a stricter validation scheme. In this research, the risk of overfitting and data leakage is reduced, and for this purpose, the grid search is introduced in addition with LOSO. This study addresses the overfitting issues commonly associated with classifiers and presents a comprehensive evaluation based on accuracy, F1 score, recall, and precision.

The classification accuracy in the patient group similarly reflects several compounding factors rather than a single argument. Patients were assisted in their movements with an exo-ankle, while healthy subjects were not using exo-ankle during data acquisition. Patients were on average almost four decades older than the healthy cohort (61 ± 12 vs. 23 ± 3 years), performed longer movement blocks (15 s vs. 5 s), and had no prior exposure to the exo-ankle device or paradigm. The patient group is also small (*n* = 7) and diagnostically heterogeneous, comprising hemiplegia, diabetic neuropathy, and ankle instability, so between-subject comparisons should be interpreted cautiously. With this caveat, we note descriptively that patients with left hemiplegia tended to achieve higher individual accuracies than those with diabetic neuropathy or ankle instability.

A study (Rahman et al., 2019) suggests that the temporal window from 10 to 15 s from the starting of stimuli shows the highest classification accuracies in all classifiers. To obtain the best-performing step size in 10 points data, a study (Hong et al., 2015) suggested that a window of 2–7 s is suggested, but these studies do not discuss the sliding windowing or overlapping windowing technique. This research examines how the sliding windowing and step of overlapping technique affect accuracy. Interestingly, the findings demonstrate that classification accuracy remained broadly stable across step sizes, with a slight increase as the number of overlapping points decreased. For example, in a simulated-online scenario, the accuracy is reported as 60.8% for an overlapping step size of a 5-point window (nearly equal to 0.5 s), which rises slightly to 61.6% for a 10-point window (1 s) and further to 62.2% for a 15-point window (1.5 s). These results indicate that accuracy increased across the three evaluated step sizes, with the largest step size (1.5 s) performing better among the three settings evaluated. In simulated-online analysis participant, 13 scored below three-class chance (23%−27%) across all step sizes (Table 4), and Table 5 shows Friedman test results for the overall comparison of the four classifiers under LOSO cross-validation. We reviewed the label timing and block alignment and found no processing error; the low accuracy likely reflects participant-level factors such as reduced optode-scalp coupling, motion during task blocks, or lower haemodynamic responsiveness, any of which are known sources of between-subject variability in fNIRS-BCI studies rather than defects in the analysis pipeline.

**Table 4:** Simulated-online accuracy by step size (leave-one-trial-out, participant-specific).

Participants	Accuracy (%) at different window step size		
	0.5 s	1 s	1.5 s
SUB 01	58.7	63.3	59.0
SUB 02	61.9	65.0	66.7
SUB 03	75.4	75.0	79.5
SUB 04	53.2	53.3	51.3
SUB 05	50.0	48.3	53.8
SUB 06	52.4	51.7	53.8
SUB 07	61.1	61.7	66.7
SUB 08	70.6	71.7	69.2
SUB 09	59.5	61.7	59.0
SUB 10	73.8	76.7	76.9
SUB 11	55.6	55.0	53.8
SUB 12	57.1	58.3	56.4
SUB 13	27.0	26.7	23.1
SUB 14	65.1	65.0	66.7
SUB 15	50.0	50.0	48.7
SUB 16	72.2	73.3	74.4
SUB 17	74.6	76.7	79.5
SUB 18	53.2	55.0	51.3
SUB 19	78.6	78.3	82.1
SUB 20	66.7	65.0	71.8
Average	60.8 ± 12.1	61.6 ± 12.5	62.2 ± 14.1

**Table 5:** Friedman test results for the overall comparison of the four classifiers under LOSO cross-validation (*n* = 20).

Performance metric	*χ^2^* (3)	*p-value*	Kendall's W
Classification accuracy	8.866	0.0311	0.143
Balanced accuracy	8.435	0.038	0.141

The equipment used in this research has a limitation of recording offline data and cannot be used for real-time implementation. So, the study was limited to in-lab simulated-online method. Real-time hardware-in-the-loop system design, testing, and validation may be implemented using independent recording sessions. To reduce computational cost, only machine learning algorithms were used, but for future prospective deep learning algorithms (Akhter et al., 2024) can be utilized to enhance the efficiency of fNIRS BCI systems. The fNIRS can be combined with other BCI modality such as EEG and EMG to create a hybrid-BCI system that may yield improved results. This BCI system is limited to three class problem dorsiflexion, plantar flexion, and rest, and this can be expanded to complete the gait cycle involving other ankle states.

A key interpretive limitation of this study concerns optode placement relative to the functional topography of the primary motor cortex. M1 is not homogeneous; the motor homunculus demonstrates a somatotopic organization in which the lower-limb representation lies on the dorsomedial surface of the precentral gyrus, extending into the interhemispheric longitudinal fissure near the vertex, corresponding approximately to the Cz and FCz regions. The 8 × 8 emitter–detector montage used in this study (AlQahtani et al., 2024) covers the major M1 area but does not include Cz and FCz. This is not merely a direction for future optimization: it directly limits our ability to attribute the classified signal specifically to lower-limb M1 activity since the recorded haemodynamic response may instead reflect adjacent cortical regions, systemic physiological changes, or other movement-related sources not localized to the foot/ankle representation. Accordingly, we present our results as evidence that a classifiable, movement-related fNIRS signal was obtained, not as confirmation that this signal specifically reflects lower-limb M1 activity. We strongly recommend that future studies reposition optodes to provide dedicated coverage of the medial/central scalp region (Cz and FCz), following montage designs specifically developed to capture lower-limb M1 activity (MacLennan et al., 2024), and include larger, multi-center multiple comparisons. Throughout this manuscript, we have accordingly framed our findings as proof-of-concept feasibility results rather than confirmed lower-limb motor-cortex decoding or a definitive clinical validation.

## 7 Conclusion

This study presents an offline and simulated-online fNIRS decoding method for prospective exo-ankle control, as a feasibility step toward future rehabilitation applications. Brain signals were acquired for three ankle states: dorsiflexion, plantar flexion, and rest. Six statistical measures, namely, mean, peak, variance, skewness, kurtosis, and slope, were analyzed as features, and four classifiers (LDA, QDA, SVM, and kNN) were compared under LOSO cross-validation. SVM achieved the highest accuracy at 72.50%, compared with 65.83% for kNN, 63.75% for LDA, and 60.42% for QDA, against a majority-class baseline of 50.0%. A Friedman test indicated a significant overall difference among classifiers (*p* = 0.0311). For the patient group, evaluated separately using SVM under the leave-one-trial-out (LOTO) cross-validation method, the mean classification accuracy was 57.23%±5.0%. A sliding window approach was used for the simulated-online implementation under participant-specific leave-one-trial-out validation using SVM, with step sizes between 0.5 and 1.5 s. Accuracy increases by the increase in step size with highest accuracy of 62.2 on 1.5-s step size. These findings demonstrate the feasibility of fNIRS-based decoding of ankle movement under participant-independent validation and provide a conservative baseline for future work toward closed-loop exo-ankle control.

## Data Availability

The datasets presented in this study can be found in online repositories. The names of the repository/repositories and accession number(s) can be found below: https://figshare.com/s/90dc2fa3fe56bd7b93de, https://doi.org/10.6084/m9.figshare.31406649.
